# Sunrise for new opportunities at ‘Carol Davila’ University of Medicine and Pharmacy

**Published:** 2010-11-25

**Authors:** F Popa

**Affiliations:** ‘Carol Davila’ University of Medicine and PharmacyRomania

The beginning of October marked a fresh start in the Romanian medical teaching system at ‘Carol Davila’ University of Medicine and Pharmacy, the host of this medical journal, with new students, opportunities and collaborations. 

The opening of the new university year had as guest of honor the respectable professor, Luigi Frati MD., Rector of ‘Sapienza’ University of Rome. He shared his wisdom with the new students encouraging them to do research in all fields,  and impressed them with the complex study of regenerative cardiology, which he presented in the event.

 On this occasion, Professor Luigi Frati has become a member of our academic community receiving the title ‘Doctor Honoris Causa’. 

**Figure 1:**
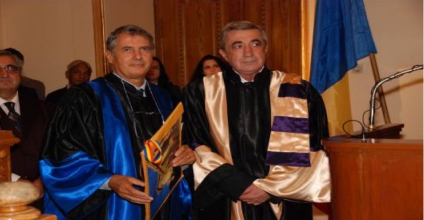


**Figure 2:**
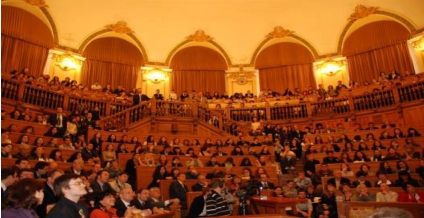


Moreover, this year marked another historical event for our university. It was consented that the honors belonging to Professor Emil Palade MD., the laureate of the Nobel Prize, should enter the patrimony of ‘Carol Davila’ University of Medicine and Pharmacy. Accordingly, his robe, medals and diplomas will be exhibited in the Aula Magna of the University starting with next year, hoping to become an inspiration for all those aiming to improve their skills and achieve more experience in their career through research and development in the medical field. This event marks the fulfillment of Professor Emil Palade's last wish, underlying the gratitude, patriotism and wisdom that the great doctors had had in the past, at the same time offering an example for next generations. 

We intensely support the ones who will always treasure work, the only value that we appreciate, the quality of work, which leads to fulfillment in medical practice, research or other related fields. I strongly believe that a new generation of youths who have adventured in this journey, which is not at all easy, will master the sacred art of medicine and through its results will have the determination and courage to honor both the university and the history.

